# Draft Genome Sequence of a Multidrug-Resistant *Klebsiella quasipneumoniae* subsp. *similipneumoniae* Isolate from a Clinical Source

**DOI:** 10.1128/genomeA.00422-16

**Published:** 2016-05-26

**Authors:** Egon A. Ozer, Andrew R. Morris, Fiorella Krapp, Christopher S. Henry, Keith E. Tyo, Wyndham W. Lathem, Alan R. Hauser

**Affiliations:** a Division of Infectious Diseases, Department of Medicine, Northwestern University, Chicago, Illinois, USA; b Department of Microbiology-Immunology, Northwestern University, Chicago, Illinois, USA; c Mathematics and Computer Science Division, Argonne National Laboratory, Lemont, Illinois, USA; d Department of Chemical and Biological Engineering, Northwestern University, Evanston, Illinois, USA

## Abstract

We report here the draft genome sequence of a multidrug-resistant clinical isolate of *Klebsiella quasipneumoniae* subsp. *similipneumoniae*, KP_Z4175. This strain, isolated as part of a hospital infection-control screening program, is resistant to multiple β-lactam antibiotics, aminoglycosides, and trimethoprim-sulfamethoxazole.

## GENOME ANNOUNCEMENT

*Klebsiella quasipneumoniae*, formerly *Klebsiella pneumoniae* phylogroup KpII, was recently taxonomically reclassified as a new sister species of *K. pneumoniae* with two subspecies, *K. quasipneumoniae* subsp. *quasipneumoniae* and *K. quasipneumoniae* subsp. *similipneumoniae* (1). *K. quasipneumoniae*, like *K. pneumoniae*, can cause human infections but is considered less pathogenic and more often associated with carriage than clinical disease (1, 2). However, severe human infections with *K. quasipneumoniae* have been reported (3, 4). Here, we report the draft genome sequence of a multidrug-resistant *K. quasipneumoniae* subsp. *similipneumoniae* strain, isolated from the gastrointestinal tract of a hospitalized patient.

*K. quasipneumoniae* subsp. *similipneumoniae* strain KP_Z4175 was isolated from a screening rectal culture obtained for infection control purposes from a 53-year-old patient. The patient had a remote history of simultaneous pancreas-kidney transplant, was currently receiving immunosuppressive treatment, and had recently undergone colectomy for an obstructing cecal lymphoma. He was admitted to a tertiary care hospital with increased ostomy output that resolved with medical management. There were no signs of active infection throughout the hospitalization. The isolate was identified as having extended-spectrum β-lactamase activity by CLSI double-disk diffusion Kirby-Bauer testing (5).

KP_Z4175 DNA was sequenced on the MiSeq platform (Illumina Inc., San Diego, CA, USA) generating 2 × 301-bp paired-end reads. A total of 15,256,306 reads were produced comprising 1,540,886,906 bases after adapter sequence trimming. *De novo* assembly was performed using SPAdes version 3.6.2 (6, 7) to generate 97 contigs at least 200 bp in length for a total sequence of 5,598,139 bp. The assembly *N*_50_ was 332,350 bp, and the average GC content was 57.6%. Annotation was performed by the NCBI Prokaryotic Genome Annotation Pipeline and contained 5,398 coding sequences. Speciation was confirmed by *fusA*, *gapA*, *gyrA*, *leuS*, and *rpoB* analysis (1) and predicted DNA-DNA hybridization of 93.7% against *K. quasipneumoniae* subsp. *similipneumoniae* strain 07A044 (accession no. CBZR00000000) using the GGDC 2.1 software (8).

To examine the antibiotic resistance profile of *K. quasipneumoniae* subsp. *similipneumoniae* strain KP_Z4175, antibiotic resistance genes were identified using ResFinder version 2.1 (9). In addition to *bla*_OKP-B-1_, a β-lactamase characteristic of *K. quasipneumoniae* (10), β-lactamase *bla*_OXA-10_ and the extended-spectrum β-lactamase *bla*_SHV-12_ were identified. Also identified were three aminoglycoside resistance genes (*aadA1*, *aacA4*, and *aac(6′)-IIc*), two fluoroquinolone resistance genes (*aac(6′)Ib-cr* and *QnrB4*), one macrolide-lincosamide-streptogramin B resistance gene (*ere(A)*), two phenicol resistance genes (*cmlA1* and *floR*), one rifampin resistance gene (*ARR-2*), two sulfonamide resistance genes (*sul1* and *sul2*), one tetracycline resistance gene (*tet(D)*), and one trimethoprim resistance gene (*dfrA14*). All identified resistance genes had nucleotide identities of 98.35 to 100% over 85 to 100% of the reference gene lengths. Broth microdilution testing using CLSI breakpoints for *Enterobacteriaceae* indicated that KP_Z4175 is resistant to gentamicin (MIC >64), tobramycin (=32), cefazolin (>64), ceftriaxone (>64), aztreonam (>64), and trimethoprim-sulfamethoxazole (>64). The isolate had intermediate resistance to ampicillin-sulbactam (=16) and pipericillin/tazobactam (=32), and was sensitive to ertapenem (≤0.03), imipenem (=1), meropenem (≤0.03), amikacin (=0.5), cefepime (=8), and ciprofloxacin (=1).

### Nucleotide sequence accession numbers.

This whole-genome shotgun project has been deposited at DDBJ/ENA/GenBank under the accession number LVCD00000000. The version described in this paper is version LVCD01000000.
